# COVID-19 Contact Tracing Apps: Predicted Uptake in the Netherlands Based on a Discrete Choice Experiment

**DOI:** 10.2196/20741

**Published:** 2020-10-09

**Authors:** Marcel Jonker, Esther de Bekker-Grob, Jorien Veldwijk, Lucas Goossens, Sterre Bour, Maureen Rutten-Van Mölken

**Affiliations:** 1 Erasmus School of Health Policy & Management Erasmus University Rotterdam Rotterdam Netherlands; 2 Erasmus Choice Modelling Centre Erasmus University Rotterdam Rotterdam Netherlands; 3 Institute for Medical Technology Assessment Erasmus University Rotterdam Rotterdam Netherlands

**Keywords:** COVID-19, discrete choice experiment, contact tracing, participatory epidemiology, participatory surveillance, app, uptake, prediction, smartphone, transmission, privacy, mobile phone

## Abstract

**Background:**

Smartphone-based contact tracing apps can contribute to reducing COVID-19 transmission rates and thereby support countries emerging from lockdowns as restrictions are gradually eased.

**Objective:**

The primary objective of our study is to determine the potential uptake of a contact tracing app in the Dutch population, depending on the characteristics of the app.

**Methods:**

A discrete choice experiment was conducted in a nationally representative sample of 900 Dutch respondents. Simulated maximum likelihood methods were used to estimate population average and individual-level preferences using a mixed logit model specification. Individual-level uptake probabilities were calculated based on the individual-level preference estimates and subsequently aggregated into the sample as well as subgroup-specific contact tracing app adoption rates.

**Results:**

The predicted app adoption rates ranged from 59.3% to 65.7% for the worst and best possible contact tracing app, respectively. The most realistic contact tracing app had a predicted adoption of 64.1%. The predicted adoption rates strongly varied by age group. For example, the adoption rates of the most realistic app ranged from 45.6% to 79.4% for people in the oldest and youngest age groups (ie, ≥75 years vs 15-34 years), respectively. Educational attainment, the presence of serious underlying health conditions, and the respondents’ stance on COVID-19 infection risks were also correlated with the predicted adoption rates but to a lesser extent.

**Conclusions:**

A secure and privacy-respecting contact tracing app with the most realistic characteristics can obtain an adoption rate as high as 64% in the Netherlands. This exceeds the target uptake of 60% that has been formulated by the Dutch government. The main challenge will be to increase the uptake among older adults, who are least inclined to install and use a COVID-19 contact tracing app.

## Introduction

The COVID-19 pandemic has formed an unprecedented public health, societal, and economic crisis. Given that no vaccine is available yet and that treatment options are limited, prevention is crucial. In an effort to stop the spread of the virus, societies have been locked down to varying degrees with social distancing; stay-at-home measures; and closures of schools, universities, and business. These policies are socially painful and economically costly.

Targeting the quarantine measures, whether enforced or voluntary, could ease the social and economic impact of these policies. Such measures require that people who are infected be quickly identified and isolated, and that their recent contacts are quarantined for the duration of the disease incubation period. Essentially, when contacts of people who are infected can be traced and quarantined at a sufficient speed, unaffected people can continue to live their lives in a more or less normal fashion. According to the World Health Organization, “when systematically applied, contact tracing will break the chains of transmission of an infectious disease and is thus an essential public health tool” [1]. There is evidence that contact tracing was effective in previous pandemics, and several model studies point to benefits in the COVID-19 crisis as well [2,3].

The main disadvantage of contact tracing is that it is labor intensive and time-consuming, and can only be effective when conducted fast. When people develop symptoms that indicate a COVID-19 infection and subsequently test positive for COVID-19, a high proportion of their contacts must be warned and quarantined quickly to avoid further infections. Manual contact tracing by public health authorities may not be able to achieve this [4]. The problem could be exacerbated if public health authorities are unable to recruit and train sufficient staff for the task.

For these reasons, the use of digital methods (ie, smartphone-based contact tracing apps) have been proposed to facilitate and accelerate contact tracing. The basic idea is that app users who have been in close proximity to someone who turned out to have been infected with COVID-19 receive a warning and are asked to self-quarantine. Such an app could theoretically replace a week’s work of manual contact tracing (per infected person) with an almost instantaneous notification once an infection has been ascertained [5].

COVID-19 contact tracing apps can only be successful in limiting the spread of the virus if a sufficient number of people are willing to download and use them. This becomes increasingly important especially when social distancing measures are relaxed and manual tracing is unable to act comprehensively and fast [5]. Based on modelling studies, the Dutch government has formulated the aim that 60% of the population should use the app [6]. It is not known whether this level of uptake can be achieved. In Singapore, it took 1 month before 20% of the population had started using the app [7], whereas in the Isle of Wight more than 40% had downloaded the contact tracing app within 10 days [8].

Dutch authorities have explicitly stated that the app will only be used for contact tracing and not to monitor or enforce self-quarantine, or to provide access to public places. However, the exact specifications of the Dutch contact tracing app have not been established yet, but they may have an impact on people’s willingness to use it. Therefore, the main aim of this study is to estimate the future uptake of a smartphone-based contact tracing app in the Dutch population and the extent that this could be optimized by changing the specifications of the app. The secondary aim is to describe differences in expected uptake between subgroups. Both aims are addressed using a discrete choice experiment (DCE).

## Methods

In a DCE, preferences for a product such as a COVID-19 contact tracing app are established by decomposing the product into separate characteristics (referred to as *attributes*) and different specifications of these characteristics (referred to as *attribute levels*) [9,10]. For example, the attribute “financial incentive” comprises the levels “€0,” “€5,” and “€10” per month (€1 = US$1.18) (see Textbox 1). The relative importance of the attribute levels is then empirically established by asking respondents to make trade-offs in a series of choice tasks. Within each choice task there are two or more products to choose from, and respondents are repeatedly asked to indicate which option they prefer. Statistical regressions are subsequently used to derive numerical values for the relative attractiveness of the attributes and its levels, using methods that have a solid foundation in random utility theory [11].

The selected attributes and attribute levels in this DCE study (see Textbox 1) reflected the Dutch context, in the sense that the Dutch government has already issued the development of a COVID-19 contact tracing app to alert users when someone they were recently near becomes infected. Currently, the launch of the app is foreseen to support a further lifting of the lockdown. However, the launch is contingent upon the app meeting the European General Data Protection Regulation privacy and safety regulations (ie, from the outset) [12]. Consequently, attributes that describe different safety and privacy levels of the app were not included in the DCE and instead were described as being part of the context of the DCE (ie, held constant across all choice tasks). The latter accommodated additional focus on the type of warnings, testing, control over the communication of a positive test result, and the potential financial incentive for app users in the DCE.

**Included attributes and levels.**

**Group size (“App users can attend…”)**

1. Activities up to 3 people
2. Activities up to 10 people
3. Activities up to 30 people
4. Activities up to 100 people

**Warning type**

1. That you were close to a person who was infected in the last 2 weeks
2. At which date and time you were close to a person who was infected

**Who is warned**

1. Only you
2. You and the local health authorities (GGD), but only with your consent
3. You and automatically the local health authorities (GGD)

**Testing after a warning**

1. Only when someone has symptoms
2. Everyone will be tested

**Who can upload test results**

1. Only you
2. This is done automatically by the government and/or local health authorities (GGD)

**Financial incentive (per month)**

1. €0
2. €5
3. €10

**Figure 1:**
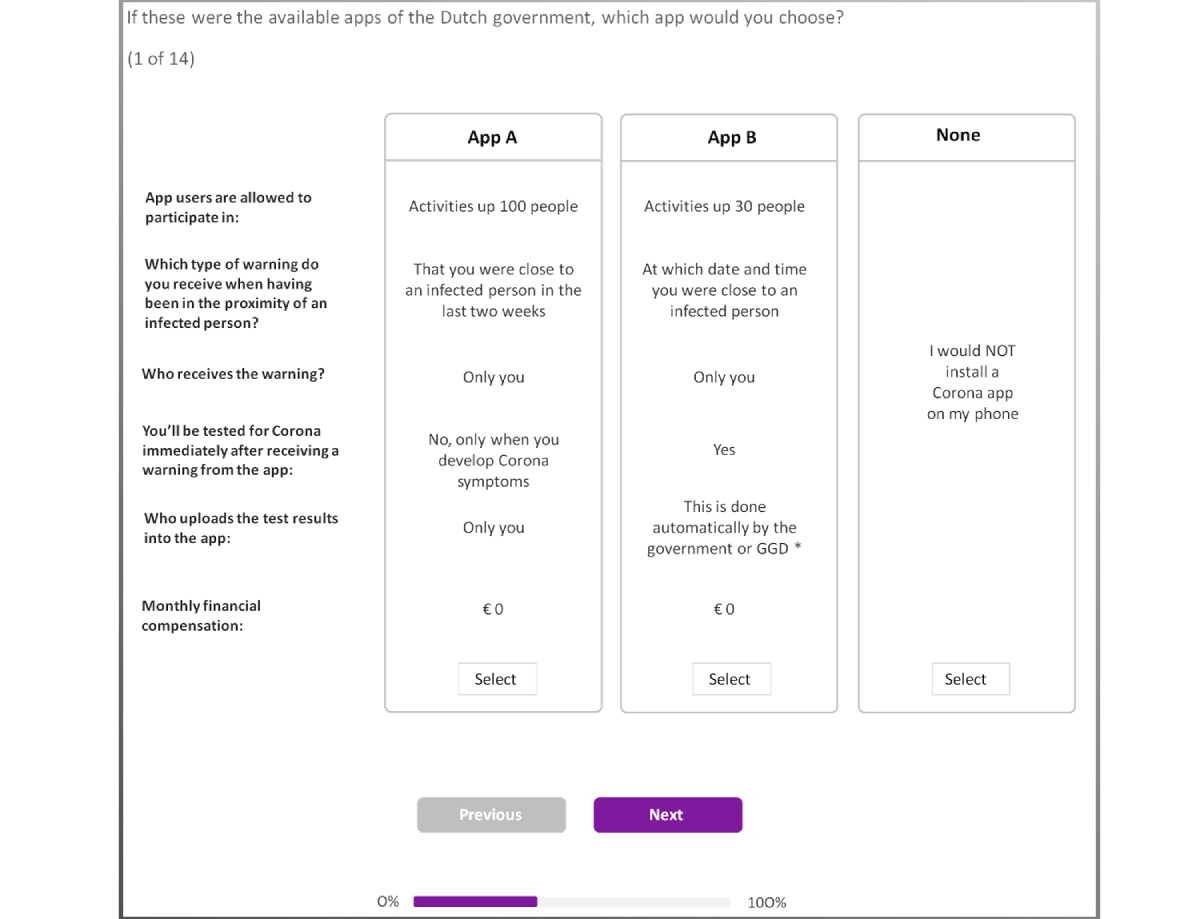
Example discrete choice task. Note: translated; original in Dutch. *GGD = local health authorities.

Once the attributes and attribute levels were defined, an initial version of the survey instrument was created using Sawtooth Software (Sawtooth Software Inc) and administered in a Dutch online panel managed by Dynata, a commercial survey sample provider. The instrument included a DCE based on a nearly orthogonal design with 300 design versions and 13 choice tasks per version. Orthogonality minimizes the correlations between attribute levels in the choice tasks and ensures statistical identification of the preference parameters. To assess the stability of the respondents’ preferences, the 14th choice task in the DCE design was a duplication of the fifth choice task [13,14]. Figure 1 provides a choice task example. As shown, respondents were able to choose between two apps and an opt out. Moreover, a small amount of attribute level overlap was used to reduce the cognitive burden of the survey and improve respondents’ attribute attendance [15]. In addition to the DCE tasks, several background, warm-up, attitudinal, and survey evaluation questions were included in the survey.

The survey instrument was pilot tested using a sample of 238 online respondents. Based on the feedback from survey participants and an evaluation of the estimated preferences, the survey instrument was revised to improve understandability and to reduce the cognitive burden of the survey. The introduction and description of the attributes were enhanced, an additional warm-up question was added, supplementary debriefing questions were included, and the levels of one of the DCE attributes (ie, the group size) were revised to better reflect the observed nonlinear preference structure. This implied that the initial data collection became incompatible with the subsequent data collection and was excluded from the final analysis.

The revised and improved instrument (available upon request) was pilot-tested using a second pilot sample of 260 participants. Based on the feedback of the respondents and our evaluation of the estimated preferences, no further changes were required. Consequently, another 640 participants were obtained to achieve an overall survey sample of 900 respondents, which was sufficient based on formal sample size calculations as well as commonly used rules of thumb [16]. The overall sample of 900 respondents was designed to be nationally representative in terms of sex, age, and educational attainment of the Dutch general population 15 years and older. All data were collected in week 16 of 2020.

Once data collection was completed, the survey satisfaction and cognitive debriefing questions were summarized by averaging the 7-point Likert scores. The survey’s dropout rates were directly observed, and completion timings were calculated as the cumulative time spent on the pages of the questionnaire, maximized at 5 minutes per page to correct for respondents taking a break in between survey questions. The stability of respondents’ preferences was assessed based on the percentage of respondents with an identical choice in repeated choice task.

Descriptive statistics were used to describe respondent characteristics for the entire sample and between subgroups that were defined based on the observed choice behavior in the DCE. More specifically, a comparison was made between respondents who always chose the app, sometimes chose the app, and never chose the app and thus always choose the opt-out option in the DCE. We examined whether the choice behavior differed by sex, age group, highest level of education, general health, chronic conditions or reduced resistance, whether someone experienced COVID-19 symptoms, and whether respondents actively used health apps. We also compared the three subgroups in terms of attitude toward the six DCE evaluation questions, the maximum group size they identified to feel comfortable with, and nine general statements related to COVID-19 tracing apps based on the Health Belief Model [17,18] that were included in the survey instrument.

Simulated maximum likelihood methods were used to estimate population- and individual-level preferences using a mixed logit (MIXL) model [19]. Such a model uses the observed choices as the dependent variable and the characteristics (ie, attribute levels) of the COVID-19 contact tracing apps shown to respondents as explanatory variables. The estimations were conducted using Stata 15 (StataCorp) with the simulated maximum likelihood calculated using 2000 Halton draws to ascertain stable coefficients and with a full variance-covariance matrix estimation aimed at accommodating potential nonzero correlations between the random parameters.

Respondents who did not choose the COVID-19 contact tracing app in any of the 14 choice tasks were excluded from the MIXL estimations and assigned to a separate (latent) class. For these respondents, it was impossible to ascertain whether they would theoretically be willing to consider installing a contact tracing app (which would merely imply a very positive opt-out parameter) or, alternatively, had lexicographic preferences and would never consider installing any contact tracing app. In the choice predictions these respondents were treated as an exogenous class with zero willingness to install and use the app. This avoided a spillover effect and upwards biased opt-out parameters of the respondents who did choose the contact tracing app in at least one of the choice tasks.

The subsequent uptake calculations were performed for three different contact tracing apps (ie, the best, worst, and most realistic app) and were based on the respondents’ individual-level preference coefficients. The best and worst contact tracing apps were defined by the MIXL estimates. The most realistic app was defined based on all publicly available information at the time of publication. For each respondent, the predicted uptake probability was calculated using the standard logit rule, after which the predicted sample adoption rate was calculated as the mean of the individual-level uptake probabilities. This is more reliable than calculations directly based on the MIXL sample mean parameters because it takes respondent heterogeneity appropriately into account. For each of the apps, the adoption rate was calculated for the entire sample of 900 respondents and for several subsamples such as different educational backgrounds (low, medium, or high) and age groups (15-34, 35-54, 55-74, ≥75 years).

## Results

### Study Population

From the total 986 panel members who started the survey and were found eligible to participate (due to quota restrictions), 900 (91.3%) completed the questionnaire, resulting in 86 dropouts (8.7%). The resulting sample was representative for the Dutch population with respect to age, sex, and education level. Of the 900 respondents, 39% (n=351) were 55 years or older, 442 (49%) respondents were male, and one-third had a lower education level (Table 1). Approximately 70% of the respondents reported that they were in good health, and 625 (69%) respondents did not have a chronic disease or a compromised immune system. Almost 25% of the respondents reported that they experienced COVID-19 symptoms during the last 2 months, and 1.4% (n=14) of respondents tested positive for COVID-19. Almost all respondents owned and used a smartphone, smartwatch, or tablet (n=827, 91.9%), and 47.60% (n=428) of respondents already used health-related apps on their mobile device. The majority of the respondents indicated that the survey was (very) interesting (n=645, 72%) and (very) clear (n=738, 82%). There were 48 (5%) respondents that found the survey (very) unclear (Table 2).

**Table 1:** Respondents’ sociodemographic characteristics for the total sample and stratified by respondents who always, sometimes, or never chose the COVID-19 app in the discrete choice experiment.^a^

Demographics		Total (N=900), n (%)	Always (n=460), n (%)	Sometimes (n=214), n (%)	Never (n=226), n (%)
**Gender**					
	Male	442 (49.1)	215 (48.6)	114 (25.8)	113 (25.6)
	Female	458 (50.9)	245 (53.5)	100 (21.8)	113 (24.7)
**Age group (years)**					
	15-34	268 (29.8)	168 (62.7)	76 (28.4)	24 (9.0)
	35-54	281 (31.2)	131 (46.6)	74 (26.3)	76 (27.0)
	55-74	265 (29.4)	124 (46.8)	59 (22.3)	82 (30.9)
	≥75	86 (9.6)	37 (43.0)	5 (5.8)	44 (51.2)
**Education level**					
	Low	274 (30.4)	134 (48.9)	51 (18.6)	89 (32.5)
	Medium	342 (38.0)	187 (54.7)	79 (23.1)	76 (22.2)
	High	284 (31.5)	139 (48.9)	84 (29.6)	61 (21.5)
**Geographical region**					
	Heavily impacted^b^	612 (68.0)	304 (49.7)	149 (24.3)	159 (26.0)
	Mildly impacted	288 (32.0)	156 (54.2)	65 (22.6)	67 (23.3)
**Self-perceived general health**					
	Good or very good	635 (70.6)	322 (50.7)	159 (25.0)	154 (24.3)
	Fair	232 (25.8)	119 (51.3)	49 (21.1)	64 (27.6)
	Bad or very bad	33 (3.7)	19 (57.6)	6 (18.2)	8 (24.2)
**Health issues**					
	Lung disease	112 (12.4)	72 (64.3)	19 (17.0)	21 (18.8)
	Heart disease	79 (8.8)	40 (50.6)	17 (21.5)	22 (27.8)
	Diabetes	87 (9.7)	44 (50.6)	13 (14.9)	30 (34.5)
	Kidney disease	11 (1.2)	7 (63.6)	2 (18.2)	2 (18.2)
	Compromised immune system	61 (6.8)	38 (62.3)	13 (21.3)	10 (16.4)
**Self-reported COVID-19 symptoms during last 2 months**					
	Yes	218 (24.2)	131 (60.1)	53 (24.3)	34 (15.6)
	No	645 (71.7)	312 (48.4)	152 (23.6)	181 (28.1)
**Tested for COVID-19 infection**					
	Yes, positive test	14 (1.6)	8 (57.1)	6 (42.9)	0 (0)
	Yes, negative test	25 (2.8)	15 (60.0)	9 (36.0)	1 (4.0)
	No	855 (95.0)	434 (50.8)	197 (23.0)	224 (26.2)
Owns and uses smartphone/smartwatch or tablet		827 (91.9)	446 (53.9)	203 (24.5)	178 (21.5)
Uses health apps on smartphone/smartwatch or tablet		428 (47.6)	272 (63.6)	100 (23.4)	56 (13.1)
**Feels comfortable around**					
	Groups of 3 people	292 (32.4)	108 (37.0)	69 (23.6)	115 (39.4)
	Groups of 10 people	312 (34.7)	196 (62.8)	66 (21.2)	50 (16.0)
	Groups of 30 people	165 (18.3)	107 (64.8)	36 (21.8)	22 (13.3)
	Groups of 100 people	79 (8.8)	34 (43.0)	31 (39.2)	14 (17.7)
	Groups of 1000 people	52 (5.8)	15 (28.8)	12 (23.1)	25 (48.1)

^a^The percentages in column 2 add up to 100% vertically, whereas the percentages in column 3-5 add up to 100% horizontally.

^b^Heavily impacted regions are Noord-Brabant, Limburg, Zuid-Holland, Noord-Holland, and Gelderland.

**Table 2:** Respondents’ attitude toward COVID-19 and evaluation of the survey for the total sample and stratified by respondents who sometimes, always, or never preferred to use the COVID-19 app.^a^

Attitudinal statements^b^			Total (N=900), n (%)	Always (n=460), n (%)	Sometimes (n=214), n (%)	Never (n=226), n (%)
**I find a contact tracing app to be useful**						
	Agree		414 (46.0)	309 (67.2)	87 (40.7)	18 (8.0)
	Disagree		191 (21.2)	30 (6.5)	33 (15.4)	128 (56.6)
**I worry about the security of a contact tracing app**						
	Agree		447 (49.7)	174 (37.8)	125 (58.4)	148 (65.5)
	Disagree		198 (22.0)	140 (30.4)	38 (17.8)	20 (8.8)
**I object to using a contact tracing app**						
	Agree		268 (29.8)	51 (11.1)	63 (29.4)	154 (68.1)
	Disagree		356 (39.6)	274 (59.6)	68 (31.8)	14 (6.2)
**I think it is very serious if I get infected with COVID-19**						
	Agree		581 (64.6)	322 (70.0)	134 (62.6)	125 (55.3)
	Disagree		91 (10.1)	38 (8.3)	30 (14.0)	23 (10.2)
**I think I would get seriously ill if I get infected with COVID-19**						
	Agree		568 (63.1)	311 (67.6)	120 (56.1)	137 (60.6)
	Disagree		119 (13.2)	60 (13.0)	38 (17.8)	21 (9.3)
**I think I have a high chance of getting infected with COVID-19**						
	Agree		198 (22.0)	123 (26.7)	46 (21.5)	29 (12.8)
	Disagree		231 (25.7)	115 (25.0)	59 (27.6)	57 (25.2)
**I think I have a high chance of getting seriously ill when infected with COVID-19**						
	Agree		374 (41.6)	214 (46.5)	78 (36.4)	82 (36.3)
	Disagree		196 (21.8)	99 (21.5)	59 (27.6)	38 (16.8)
**I think a COVID-19 app is a good way to control and fight COVID-19**						
	Agree		416 (46.2)	312 (67.8)	84 (39.3)	20 (8.8)
	Disagree		222 (24.7)	41 (8.9)	52 (24.3)	129 (57.1)
**I would use a contact tracing app if it becomes available**						
	Agree		388 (43.1)	307 (66.7)	72 (33.6)	9 (4.0)
	Disagree		250 (27.8)	30 (6.5)	56 (26.2)	164 (72.6)
**Survey evaluation**						
	**The choice questions were clear**					
		Agree	738 (82.0)	385 (83.7)	172 (80.4)	181 (80.1)
		Disagree	48 (5.3)	23 (5.0)	16 (7.5)	9 (4.0)
	**The choice questions were interesting**					
		Agree	645 (71.7)	373 (81.1)	161 (75.2)	111 (49.1)
		Disagree	70 (7.8)	23 (5.0)	17 (7.9)	30 (13.3)
	**I could easily recognize the differences between the apps in the choice questions**					
		Agree	667 (74.1)	360 (78.3)	158 (73.8)	149 (65.9)
		Disagree	76 (8.4)	39 (8.5)	21 (9.8)	16 (7.1)
	**I could easily choose between the apps in the choice questions**					
		Agree	633 (70.3)	332 (72.2)	154 (72.0)	147 (65.0)
		Disagree	101 (11.2)	59 (12.8)	27 (12.6)	15 (6.6)
	**I could easily have answered more choice questions**					
		Agree	617 (68.6)	331 (72.0)	152 (71.0)	134 (59.3)
		Disagree	50 (5.6)	25 (5.4)	13 (6.1)	12 (5.3)
	**There were too many choice questions**					
		Agree	174 (19.3)	90 (19.6)	40 (18.7)	44 (19.5)
		Disagree	469 (52.1)	259 (56.3)	115 (53.7)	95 (42.0)

^a^The percentages in columns 2-5 add up to 100% vertically, but in columns 3-5, 100% is the amount of people in that specific group.

^b^Reported on respondents who completely agreed or agreed and who completely disagreed or disagreed; percentages do not count up to 100%, as respondents who answered *neutral* were not included in this table.

### DCE Results

All of the contact tracing app attributes influenced respondents’ preferences (Table 3). On average, respondents preferred a COVID-19 contact tracing app that offers them additional benefits in terms of a small financial reward of €5 or €10 a month, being allowed to meet with groups of up to 10 or 30 people, and being tested if they were near a person who was infected. On average, respondents wanted to remain in charge of their own data by giving explicit permission to share the alert with the local health authorities (GGD) and entering a positive COVID-19 test result into the app themselves. They preferred alerts that are specific with respect to date and time.

**Table 3:** Mixed logit estimation results.

Attributes		Population mean (SE)	95% CI	Population SD (SE)	95% CI
No app		–3.44 (0.32)	–4.07 to –2.80	3.97 (0.28)	3.43 to 4.51
**Group size**					
	3 people (reference)	0	N/A^a^	0	N/A
	10 people	0.56 (0.09)	0.39 to 0.74	1.27 (0.14)	1.01 to 1.55
	30 people	0.45 (0.10)	0.25 to 0.65	1.77 (0.13)	1.51 to 2.03
	100 people	0.04 (0.12)	­–0.20 to 0.28	2.50 (0.17)	12.17 to 2.84
**Warning type**					
	Limited information (reference)	0	N/A	0	N/A
	Detailed information	0.23 (0.06)	0.10 to 0.36	1.14 (0.09)	0.97 to 1.31
**Who is warned**					
	Only you (reference)	0	N/A	0	N/A
	You and automatically the local health authorities	0.01 (0.07)	–0.12 to 0.15	1.02 (0.09)	0.83 to 1.20
	You and local health authorities after your consent	0.28 (0.07)	0.14 to 0.42	0.94 (0.09)	0.74 to 1.13
**Testing after a warning**					
	Only when someone has symptoms (reference)	0	N/A	0	N/A
	Everyone	0.40 (0.09)	0.23 to 0.57	1.96 (0.10)	1.77 to 2.15
**Who can upload test results**					
	You (reference)	0	N/A	0	N/A
	Local health authorities	0.05 (0.08)	–0.11 to 0.20	1.70 (0.08)	1.53 to 1.87
**Financial incentive (€ per month)**					
	0 (reference)	0	N/A	0	N/A
	5	0.85 (0.11)	0.62 to 1.07	2.44 (0.13)	2.18 to 2.70
	10	1.29 (0.16)	0.97 to 1.60	3.70 (0.18)	3.35 to 4.05

^a^N/A: not applicable.

The attribute levels had the expected sign (showing theoretical validity), and 84% (760/900) of the respondents showed consistency in their choices (ie, they opted for the same alternative in the fifth and 14th DCE choice tasks). The utility pattern for the attribute *group size* was hyperbolic (ie, respondents preferred an app that allowed them to meet with 10 or 30 individuals instead of 3 individuals but were less positive about meeting with 100 individuals; Table 3). Furthermore, on average and relative to the other attributes, financial incentive was the most important attribute, while the attribute describing who enters into the app that the app user has tested positive was the least important attribute. However, the standard deviations of the alternative specific constant (ie, random intercept) and all attribute (levels) indicated a wide variation in preferences among respondents.

### COVID-19 Contact Tracing App Uptake

Over half of the respondents (460/900, 51%) chose a contact tracing app in all choice tasks, which means that they preferred a contact tracing app with the least preferred specifications over no contact tracing app at all (see Table 1). About 25% (226/900) of the respondents had strict preferences against a contact tracing app (ie, they chose the opt-out alternative in all 14 DCE tasks) and could not be persuaded to choose a contact tracing app, not even the app with the most preferred specifications. The choices of the remaining 24% (214/900) of the respondents depended on the specifications of the app.

**Figure 2:**
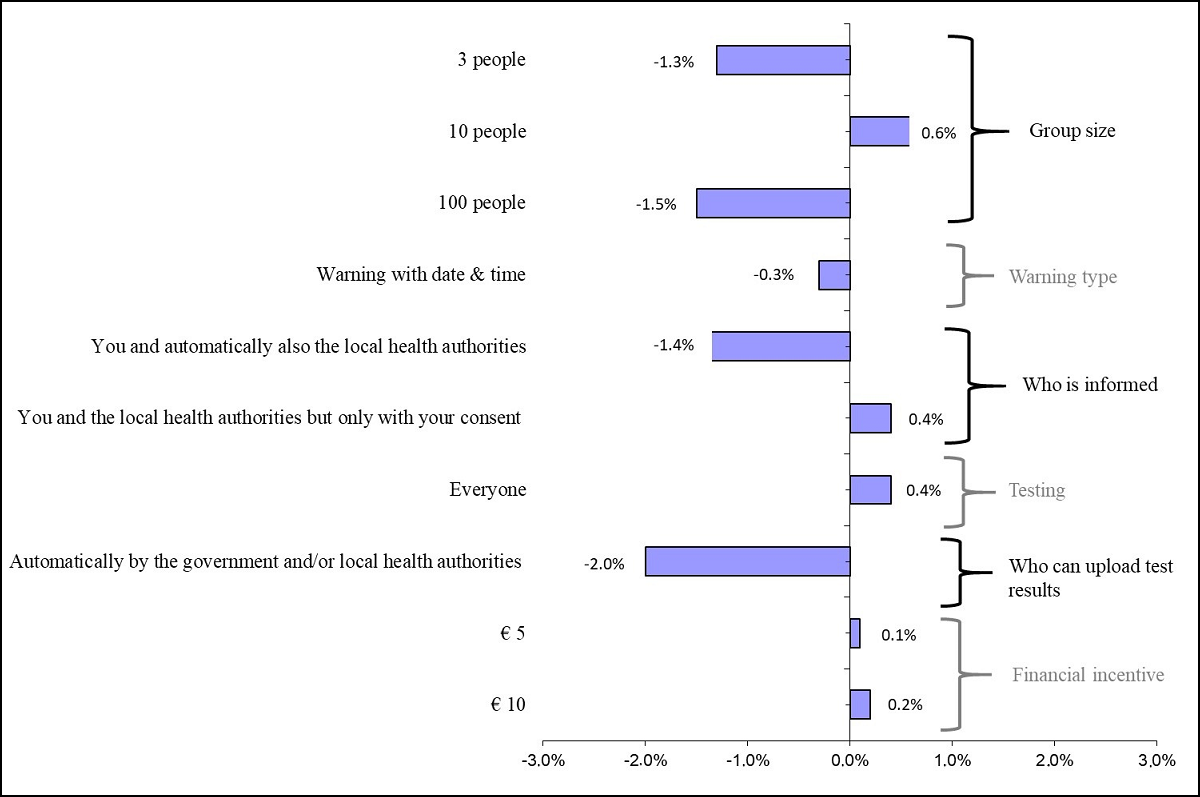
Univariate marginal estimates for increase in predicted adoption rate; attributes level changes vs base case. Note: The base case is the most realistic COVID-19 contact tracing app that allows the user to meet with 30 individuals, warns the user that they were close to a person who was infected in the last 2 weeks, warns the user and the local health authorities (GGD), allows the user to undergo a COVID-19 test only after they have COVID-19 symptoms, is updated by the local health authorities (GGD) that the user tested positive for COVID-19, and does not give the user a financial incentive. This base case is indicated as zero change in the probability of the x-axis.

Assuming that the most realistic COVID-19 contact tracing app, given the situation in the Netherlands at the time of writing, is defined by an app that allows the app user to meet with 30 individuals at the same time, warns the app user that they were close to a person who was infected in the last 2 weeks, warns only the app user, allows the app user to undergo a COVID-19 test only after they has COVID-19 symptoms, is updated by the app user that they tested positive for COVID-19, and does not give the app user a financial incentive, the predicted adoption rate of the most realistic app was 64.1% (Table 4). One-way changes in our app’s attribute levels had a relatively small impact on the predicted adoption rate (Figure 2). Changing from a contact tracing app with the least preferred to the most preferred attribute levels, the estimated adoption rate of the contact tracing app for the Dutch population increased from 59.3% to 65.7%. It should be noted that such changes do not perfectly correlate with the MIXL mean preference parameters; the degree of preference heterogeneity is an equally important determinant.

There are important sociodemographic differences in predicted adoption rates. Survey respondents aged between 15 and 34 years were more likely to use a contact tracing app than people 75 years or older. Survey respondents younger than 35 years were also more sensitive to the specifications of the app. When comparing the contact tracing app with the least preferred to the app with the most preferred specifications, the adoption rates increased from 72.4% to 81.7% for people younger than 35 years and decreased from 46.4% to 45.6% for people 75 years or older. The predicted adoption rates also differed by educational attainment. Survey respondents with lower levels of education were less likely to install the app and less sensitive to the specifications of the app. When comparing the least and most preferred contact tracing app, the adoption rates increased from 55.4% to 59.1% for the lowest educated respondents and from 59.4% to 67.8% for the highest educated respondents. Furthermore, as general health worsened, the proportion of respondents that always preferred a contact tracing app increased. That proportion was also higher among respondents with a lung disease, a kidney disease, and a compromised immune system compared to respondents without health problems.

We also observed important attitudinal differences in adoption. Respondents who indicated feeling safe in large groups (up to 1000 people), considered the chance of being infected with COVID-19 to be small, and did not think they would become seriously ill when infected by COVID-19 were more likely to reject the app irrespective of its specifications. That also holds for respondents who were more worried about the security of the app.

Besides the attributes included in this study, frequently mentioned reasons that favor the use of a COVID-19 contact tracing app were prevention (being able to control the virus), uncertainty reduction (ie, clarity and security), and more freedom. Frequently mentioned barriers were related to privacy concerns, safety concerns (data leaks), not owning a smartphone, potentially required out-of-pocket costs, and a low expected adoption rate in the society.

**Table 4:** Predicted COVID-19 contact tracing app adoption rates (%), stratified by age and education level.

Apps	All (n=900), %	15-34 years (n=268), %	35-54 years (n=281), %	55-74 years (n=265), %	≥75 years (n=86), %	Low educ^a^ (n=274), %	Medium educ (n=342), %	High educ (n=284), %
Most preferred app^b^	65.7	81.7	61.4	60.4	46.0	59.1	69.2	67.8
Most realistic app^c^	64.1	79.4	60.4	58.4	45.6	59.3	67.1	65.0
Least preferred app^d^	59.3	72.4	55.4	54.3	46.4	55.4	62.3	59.4

^a^educ: education.

^b^Specifications of the most preferred COVID-19 contact tracing app were the app user is allowed to meet with 10 individuals at the same time, warns the app user that they were close to a person who was infected in the last 2 weeks, warns the app user and the local health authorities (GGD) after permission, allows the app user to undergo a COVID-19 test, is updated by the app user that they tested positive for COVID-19, and does give the app user a financial incentive of €10 per month.

^c^Specifications of the most realistic app were allows the app user to meet with 30 individuals at the same time, warns the app user that they were close to a person who was infected in the last 2 weeks, warns the app user, allows the app user to undergo a COVID-19 test only after they have COVID-19 symptoms, is updated by the app user that they tested positive for COVID-19, and does not give the app user a financial incentive.

^d^Specifications of the least preferred app were allows the app user to meet with 3 individuals at the same time, warns the app user the date and time that they were close to a person who was infected, warns the local health authorities (GGD), allows the app user to undergo a COVID-19 test only after they have COVID-19 symptoms, is updated by the local health authorities (GGD) that the app user tested positive for COVID-19, and does not give the app user a financial incentive.

## Discussion

### Main Findings

Our study suggests that an adoption rate as high as 66% can be achieved for a contact tracing COVID-19 app in the Netherlands. However, there is wide variation in preferences. Over half of the respondents always chose to use an app, about 25% of the respondents could never be persuaded to choose an app, and the choice of the remaining 24% of the respondents depended on the specifications of the app. Changing the specifications from the least to the most preferred increased the predicted adoption rate from 59% to 66% in the entire sample. In general, app users prefer an app that offers them additional benefits in terms of being allowed to meet in groups of up to 10 and 30 people, and being tested immediately after the alert that they were near a person who was infected. App users want to remain in charge of their own data by giving explicit permission to share the alert with the public health authorities and entering a positive test result into the app themselves. They prefer alerts that are specific with respect to date and time. A small financial reward of €5 or €10 a month is appreciated.

### Policy Context and Implications

The presented results should be viewed in the context of the discussions about a COVID-19 app in the Netherlands up until mid-April, when the data were collected. In the Netherlands, the peak in the number of patients with COVID-19 in hospital intensive care units was reached in the first week of April, and the curve was at the beginning of a decline, which was not yet clear at that time. Test capacity was limited and only available for individuals with severe symptoms and hospital staff.

In mid-April, the Dutch government organized a 2-day long *appathon* to review and test 7 different candidate apps that were selected from a long list of 660 proposed apps. The appathon was broadcasted on the internet. It turned out that the candidate apps all had privacy and security issues. Consequently, none of the apps in the appathon were selected and the Ministry of Health initiated the development of a new app, which would, from the outset, be designed with strict privacy and security in mind.

This governmental decision confirms that we took the right choice context for our study, namely, that the proposed app would meet the required privacy and data security issues instead of asking respondents to trade off privacy and security for benefits or specifications of the app. The Dutch authorities also made it clear that it would not adopt a contact tracing app that stored location data and that contact data should not be stored for longer than 2-3 weeks, which concurs with our decision not to include location data and length of data storage in the trade-offs either. In the literature, smartphone apps that seem to meet these conditions have been presented [12,20], and almost all new apps that appear in the continuously updated database of the Massachusetts Institute of Technology, which captures details of every significant automated contact tracing effort around the world, are based on the relatively secure and privacy respecting Bluetooth application programming interface, as introduced by Google and Apple [21].

Our finding that the adoption rate of the most realistic app was 34% points higher for respondents aged 15-34 years than for respondents 75 years or older may have policy implications. It suggests the need for a tailored communication strategy to maximize the uptake of the contact tracing app. Our data indicated that older adults felt less comfortable in larger groups and were more anxious about getting infected and getting seriously ill when infected, which is logical given the higher prevalence of health problems among older adults and their greater susceptibility to COVID-19. If this indicates that older adults would feel insufficiently protected by a contact tracing app, this may have contributed to the lower adoption rate among older adults. A tailored communication strategy should address these concerns and convince older adults of the necessity to share data to control a virus that largely spreads asymptomatically even if the app does not provide individual protection. Because people younger than 35 years were more sensitive to the specifications of the app they can be tempted to adopt the app by communicating the benefits to the app user, such as being allowed to meet in larger groups, immediately getting tested after contact with a person who was infected with COVID-19, and perhaps a financial reward.

Appropriately addressing the observed attitudinal differences toward adopting the app is another challenge for policy makers. Perhaps the group that feels safe in large groups of app users, thinks the chance is small they will get the virus, and does not think that they will become seriously ill if they are infected by the virus represents a group that downplays the seriousness of the situation. Education that is specifically tailored to these attitudes might be necessary.

### Comparison With Other Studies

The context that respondents were offered in this study (ie, that the app would comply with privacy and security legislations) is likely to have contributed to the high adoption rates. Nevertheless, the only other choice-based study about COVID-19 apps published so far has reported even higher adoption rates, despite the fact that they did include attributes like using the app to enforce self-isolation, anonymity, length of data storage, and responsibility for the app project [22]. In this UK-wide study, the app with recommended specifications had a 73.5% adoption rate compared with 64.1% in our study. One of the possible reasons could be that the study in the United Kingdom was done earlier, when the infection peak had not yet been reached and people felt more insecure.

There have been several other surveys about COVID-19 apps, but these were not choice based and did not ask respondents to trade-off positive versus negative characteristics of an app, as is done in a DCE. In a large international survey conducted in France, Germany, Italy, the United Kingdom, and the United States, strong support for a contact tracing app was found regardless of the respondents’ country or background characteristics [23].

### Limitations

The study was conducted in a representative sample of the Dutch population with respect to age, gender, and education. Nevertheless, we should acknowledge that respondents were members of an internet panel of a market research organization, which makes them more likely to have a positive attitude toward internet and digital devices in general and thus more likely to adopt an app. This is related to our finding that people who already use health apps were more likely to prefer the contact tracing app than people who do not use health apps. However, the impact of using an internet panel is probably limited, as 88% of Dutch citizens owns a smartphone and over one-third has a health app installed on it [24].

It is obvious that the adoption rates in our study are based on stated preferences, which might differ from revealed preferences. First, although stated preferences may accurately reflect an individual’s intention to use an app, they may not accurately predict real-world use of an app [25]. There are few external validation studies of DCEs, but there are cases such as influenza vaccination and colorectal cancer screening in which over 90% of choices were correctly predicted at an individual level [25,26]. However, one may argue that there are less privacy and security issues involved in these cases. Second, the presented analyses do not take dynamics into account and thus only predict the potential uptake of a contact tracing app and not the time it takes for the predicted uptake to be achieved. The latter likely depends on the attractiveness of the app but also on external factors, including the amount of effort from local health authorities and the government to promote the contact tracing app using public health campaigns. Third, the achieved adoption rates of the contact tracing app will likely depend on the timing of its launch. If people still recognize the seriousness of the COVID-19 pandemic and the necessity of a contact tracing app as they did in our study (based on mid-April 2020 data collection), they may be willing to cooperate and share personal data more easily than if they view the crises as being defeated. With COVID-19 restrictions currently being eased, it seems conceivable that respondent preferences could change accordingly.

### Conclusion

Based on the presented results, with predicted app adoption rates ranging from 59% to 66%, we conclude that it is possible for a secure and privacy-respecting COVID-19 contact tracing app to reach a high adoption rate. Taking account of the preferred specifications of the app will contribute to a more widespread adoption. The main challenge will be to increase the adoption rate among older adults (≥75 years of age), since even the app with the most preferred characteristics had a 36%-point lower adoption rate compared to respondents 35 years and younger.

## Appendix

Multimedia Appendix 1 Cherries checklist.

## Abbreviations

DCE: discrete choice experiment
MIXL: mixed logit
