# Supplementary material for: COVID-19 Contact Tracing Apps: Predicted Uptake in the Netherlands Based on a Discrete Choice Experiment
Source: JMIR Mhealth Uhealth. 2020 Oct 9;8(10):e20741. doi: 10.2196/20741 (PMC7584977; doi:10.2196/20741)
Supplement: Multimedia Appendix 1 [file mhealth_v8i10e20741_app1.pdf]

Item Category	Checklist Item	Explanation & Answer
**Design**		
	Describe survey design	Describe target population, sample frame. Is the sample a convenience sample? (In “open” surveys this is most likely.)<br><br>**The survey was administered in an online panel managed by Dynata, a well-known survey sample provider. The target population was the Dutch general population (18 years and older). Representativeness of the sample was ensured via “age-sex-educ” quota sampling brackets, i.e. with 24 stratification brackets in total.**
**IRB (Institutional Review Board) approval and informed consent process**		
	IRB approval	**No, the survey was not IRB approved; in the Netherlands this is not required for stated preference / discrete choice surveys that target the general population.**
	Informed consent	Describe the informed consent process. Where were the participants told the length of time of the survey, which data were stored and where and for how long, who the investigator was, and the purpose of the study?<br><br>**The informed consent was part of the survey. Respondents were told the length of the survey (15 minutes), that all data would be confidential, only be used for academic research, and not be shared with third parties.**
	Data protection	If any personal information was collected or stored, describe what mechanisms were used to protect unauthorized access.<br><br>**The survey was anonymous; personal identifiable information was not collected and/or stored.**
**Development and pre-testing**		
	Development and testing	State how the survey was developed, including whether the usability and technical functionality of the electronic questionnaire had been tested before fielding the questionnaire.<br><br>**The survey was developed using well-tested commercial survey administration software (Sawtooth Software) that has build-in support for the type of discrete choice survey questions, randomizations, and quota controls that we needed for this particular survey. As such, our pilot tests were not aimed at testing the technical functionality of the survey but instead on the overall flow, visual layout, correct specification of the quota controls, and on the wording of the questions. The survey was tested by all team members and colleagues prior to the data collection.**
**Recruitment process and description of the sample having access to the questionnaire**		

	Open survey versus closed survey	An “open survey” is a survey open for each visitor of a site, while a closed survey is only open to a sample which the investigator knows (password-protected survey).<br><br>**Targeted invitations were sent out in small batches by the survey sample provider until each of the 24 quota brackets were filled.**
	Contact mode	Indicate whether or not the initial contact with the potential participants was made on the Internet. (Investigators may also send out questionnaires by mail and allow for Web-based data entry.)<br><br>**Participants are panel members and invited by the survey sample provider to participate in the surveys. We had no initial contact with the respondents.**
	Advertising the survey	How/where was the survey announced or advertised? Some examples are offline media (newspapers), or online (mailing lists – If yes, which ones?) or banner ads (Where were these banner ads posted and what did they look like?). It is important to know the wording of the announcement as it will heavily influence who chooses to participate. Ideally the survey announcement should be published as an appendix.<br><br>**The survey was not advertised.**
**Survey administration**		
	Web/E-mail	State the type of e-survey (eg, one posted on a Web site, or one sent out through e-mail). If it is an e-mail survey, were the responses entered manually into a database, or was there an automatic method for capturing responses?<br><br>**We used a website-based survey.**
	Context	Describe the Web site (for mailing list/newsgroup) in which the survey was posted. What is the Web site about, who is visiting it, what are visitors normally looking for? Discuss to what degree the content of the Web site could pre-select the sample or influence the results. For example, a survey about vaccination on a anti-immunization Web site will have different results from a Web survey conducted on a government Web site<br><br>**The survey was published on a subdomain of the survey administration software website (http://www.sawtoothsoftware.com)**
	Mandatory/voluntary	Was it a mandatory survey to be filled in by every visitor who wanted to enter the Web site, or was it a voluntary survey?<br><br>**Voluntary.**
	Incentives	Were any incentives offered (eg, monetary, prizes, or non-monetary incentives such as an offer to provide the survey results)?<br><br>**Yes, panelists receive a small financial compensation for filling out surveys.**
	Time/Date	In what timeframe were the data collected?<br><br>**The data were collected over the course of 2 days.**
	Randomization of items or questionnaires	To prevent biases items can be randomized or alternated.<br><br>**The order of the survey questions and response options was fixed but**

		**there were 300 different design versions in the discrete choice experiment. Each respondent only received 1 version to complete.**
	Adaptive questioning	Use adaptive questioning (certain items, or only conditionally displayed based on responses to other items) to reduce number and complexity of the questions.<br><br>**Adaptive questions were not used.**
	Number of Items	What was the number of questionnaire items per page? The number of items is an important factor for the completion rate.<br><br>**Typically one question per page. Short questions (such as age, sex) were combined on a single page and the final evaluation questions were included in a grid-based style with Likert response options.**
	Number of screens (pages)	Over how many pages was the questionnaire distributed? The number of items is an important factor for the completion rate.<br><br>**41 pages (including information pages and warm-up questions)**
	Completeness check	It is technically possible to do consistency or completeness checks before the questionnaire is submitted. Was this done, and if "yes", how (usually JAVAScript)? An alternative is to check for completeness after the questionnaire has been submitted (and highlight mandatory items). If this has been done, it should be reported. All items should provide a non-response option such as "not applicable" or "rather not say", and selection of one response option should be enforced.<br><br>**Most questions were designated as 'require response'. For these questions, the survey administration system validates the provided answers (using Javascript and server-side validation) when the respondent clicks on 'next' to proceed to the next survey page.**
	Review step	State whether respondents were able to review and change their answers (eg, through a Back button or a Review step which displays a summary of the responses and asks the respondents if they are correct).<br><br>**Yes, respondents were able to go backwards in the survey and allowed to revise their previous answers until the survey was officially submitted.**
**Response rates**		
	Unique site visitor	If you provide view rates or participation rates, you need to define how you determined a unique visitor. There are different techniques available, based on IP addresses or cookies or both.<br><br>**The survey sample provider ensured the `uniqueness' of our respondents. Within our survey, respondents received a unique respondent identification code by the survey administration system.**
	View rate (Ratio of unique survey visitors/unique site visitors)	Requires counting unique visitors to the first page of the survey, divided by the number of unique site visitors (not page views!). It is not unusual to have view rates of less than 0.1 % if the survey is voluntary.<br><br>**Not applicable.**
	Participation rate (Ratio of unique visitors who agreed to participate/unique first survey page visitors)	Count the unique number of people who filled in the first survey page (or agreed to participate, for example by checking a checkbox), divided by visitors who visit the first page of the survey (or the informed consents page, if present). This can also be called "recruitment" rate.<br><br>**Of those respondents who entered the survey (N=1115), 46 did not fill out any screening questions, 8 dropped while filling out age, sex, or education level background info, 74 were screened-out by the survey administration system because specific survey quota brackets were**

		**already full.**
	Completion rate (Ratio of users who finished the survey/users who agreed to participate)	The number of people submitting the last questionnaire page, divided by the number of people who agreed to participate (or submitted the first survey page). This is only relevant if there is a separate "informed consent" page or if the survey goes over several pages. This is a measure for attrition. Note that "completion" can involve leaving questionnaire items blank. This is not a measure for how completely questionnaires were filled in. (If you need a measure for this, use the word "completeness rate".)<br><br>**Of the respondents (N=987) who started with the survey, 18 dropped out before the DCE questions, 19 during the DCE warm-up questions, 22 during the main DCE questions, and 28 after completing the DCE, resulting in an overall sample of N=900 completes.**
**Preventing multiple entries from the same individual**		
	Cookies used	Indicate whether cookies were used to assign a unique user identifier to each client computer. If so, mention the page on which the cookie was set and read, and how long the cookie was valid. Were duplicate entries avoided by preventing users access to the survey twice; or were duplicate database entries having the same user ID eliminated before analysis? In the latter case, which entries were kept for analysis (eg, the first entry or the most recent)?<br><br>**The survey sample provider ensured unique respondents and provided unique person identification codes that were stored as a pass-in variable in our database. Before the final analysis, the uniqueness of the person identification codes was confirmed.**<br><br>**Users could only log in (once) via the survey sample provider and received a cookie that stored their current progress in the survey. This allowed respondents to pause and resume the survey even if the browser had been closed. After the user completed the survey, it was no longer possible to make changes or re-submit answers.**
	IP check	Indicate whether the IP address of the client computer was used to identify potential duplicate entries from the same user. If so, mention the period of time for which no two entries from the same IP address were allowed (eg, 24 hours). Were duplicate entries avoided by preventing users with the same IP address access to the survey twice; or were duplicate database entries having the same IP address within a given period of time eliminated before analysis? If the latter, which entries were kept for analysis (eg, the first entry or the most recent)?<br><br>**No IP address checks were necessary (nor possible, since IP addresses were not stored due to privacy concerns)**
	Log file analysis	Indicate whether other techniques to analyze the log file for identification of multiple entries were used. If so, please describe.<br><br>**No other checks for multiple entries were conducted.**
	Registration	In "closed" (non-open) surveys, users need to login first and it is easier to prevent duplicate entries from the same user. Describe how this was done. For example, was the survey never displayed a second time once the user had filled it in, or was the username stored together with the survey results and later eliminated? If the latter, which entries were kept for analysis (eg, the first entry or the most recent)?

		**Not applicable.**
**Analysis**		
	Handling of incomplete questionnaires	Were only completed questionnaires analyzed? Were questionnaires which terminated early (where, for example, users did not go through all questionnaire pages) also analyzed?<br><br>**Incomplete surveys were not analyzed.**
	Questionnaires submitted with an atypical timestamp	Some investigators may measure the time people needed to fill in a questionnaire and exclude questionnaires that were submitted too soon. Specify the timeframe that was used as a cut-off point, and describe how this point was determined.<br><br>**Time stamps were stored but not used to exclude respondents.**
	Statistical correction	Indicate whether any methods such as weighting of items or propensity scores have been used to adjust for the non-representative sample; if so, please describe the methods.<br><br>**Such methods were not used in the statistical analyses.**
